# Reply to the Reviewers of Mr. Bampfield's Paper on Variola and Varicella

**Published:** 1822-09

**Authors:** R. W. Bampfield

**Affiliations:** One of the Surgeons to the Royal Metropolitan Infirmary for Diseases of Children, &c.


ART. IV.—*Reply to the Reviewers of Mr. Bampfield's Paper on Variola and Varicella.* By R. W. BAMPFIELD, Esq. one of the Surgeons to the Royal Metropolitan Infirmary for Diseases of Children, &c.

WHEN I wrote the history of the cases of varicella and variola for this Journal, it was not expected they would have been honoured by the criticisms of the editor of the Medico-Chirurgical Review, or the writer of the Historical Essay on the State of the Medical Sciences in this Journal. It is a singular coincidence that both have attributed to me the same opinions and conclusions, although in that paper I hazarded no opinion, nor did I venture, from such solitary facts, to draw any conclusion! and, although I endeavoured to be clear and perspicuous, still I am bound, by the laws of politeness, to suppose I did not express myself so as to be sufficiently understood, or both would not have imputed to me the opinion that variola and varicella are diseases of identity; for, on reference to that paper, it will be found that the word identity was only *twice* employed by me, and the use of it was each time in the phrase "*identity of origin*:"—1st, in assigning as a reason for submitting the history of the cases to the public, that "it was strictly applicable to the interesting question now agitated by the profession respecting the *identity of origin* of chicken and small pox;" and, 2dly, in stating, "it appeared to me the cases gave some little additional weight to the opinions of Dr. Macleod, Dr. Thomson, Messrs. Cross, Black, &c. in favour of the *identity of origin* of variola and varicella."

Of the identity of origin some further evidence appears in Dr. Reed's paper in the Edinburgh Medical Journal for April; and it seems clear that the opinions stated to be espoused by the above gentlemen, and which, I thought, my cases favoured, relate not to the "identity of variola and varicella," or to the identity of those diseases, but to the identity of their origin: and, surely, it will not be difficult to the medical mind to admit that diseases, differing in character, have an identity of origin; for, by the consentaneous opinion or belief of the medical world, many fevers, differing in characteristic symptoms, are allowed to derive an identity of origin from marsh miasma; and the whole class of phlegmasiæ have an identity of origin in cold or checked perspiration; so that the admission of the truth of these two examples would render it unnecessary to prosecute this argument further.

Both my reviewers have assumed and concluded that my cases of varicella were modified small-pox. This "argumentum ad ignorantiam" seems uncourteous, and rather unhappily chosen; for it certainly militates both against their assumption and conclusion, that varicella existed before small-pox *modified by vaccination* was observed, and was universally considered a distinct disease. That I am an old member of the profession, and had opportunities, during my pupilage to gentlemen who attended one of the largest infirmaries in London, of witnessing and of distinguishing varicella before vaccination was practised, and many years before the modified small-pox from it was noticed at all; and as I now attend the Royal Metropolitan Infirmary for Sick Children, where the cases of eruptive diseases are very numerous, it is trusted I may, without much presumption, assume that I, who saw the cases daily, ought to know whether they appertained to varicella or modified small-pox, at least as well as my reviewers who did not see them.

The distinction between the eruption of variola and varicella has been pointed out by Dr. Allison, Mr. Cross, and all the gentlemen I have quoted, besides others; and the difference between modified small-pox and varicella is equally clear, for the eruption of modified small-pox is the same as variola that runs its usual course; but the eruption of the modified small-pox fills, breaks, crusts, and falls off, three or four days sooner than that of the regular small-pox, and is rarely followed by secondary fever. In the cases of modified small-pox after vaccination first observed, as of Miss Martin recorded by Mr. Tegart, "Dr. Heberden hesitated but little in pronouncing the disease to be small-pox;" yet its duration was shortened, and there was no secondary fever. See also the case of Miss Reynholds, in the same Medical and Physical Journal for September 1811; and the reports from Crediton, &c. in other Numbers of the Journal. The matter of modified small-pox will, by inoculation, produce regular variola; the *lymph* of varicella will not.

It may be asked, what advantage is to be derived from the knowledge of variola and varicella having an identity of origin, if the fact be finally established, and of the one producing the other by its contagious miasma? The answer is, that it will enable the profession to guard the unvaccinated against small-pox by proper prophylactic means.

In conclusion, I would observe that there are some facts and arguments that favour the opinion of identity of disease; but, to my mind, they are not satisfactory evidence of the fact.

*37, Bedford-street, Covent Garden; July 13th, 1822.*

